# A Q-analysis package for higher-order interactions analysis in Python and its application in network physiology

**DOI:** 10.3389/fnetp.2025.1691159

**Published:** 2025-10-29

**Authors:** Nikita Smirnov, Semen Kurkin, Alexander E. Hramov

**Affiliations:** ^1^ Baltic Center for Neurotechnology and Artificial Intelligence, Immanuel Kant Baltic Federal University, Kaliningrad, Russia; ^2^ Research Institute for Applied Artificial Intelligence and Digital Solutions, Plekhanov Russian University of Economics, Moscow, Russia

**Keywords:** Q-analysis, complex networks, higher-order interactions, network physiology, functional networks, network topology, simplex

## Abstract

**Introduction:**

Real-world networks possess complex, higher-order structures that are not captured by traditional pairwise analysis methods. Q-analysis provides a powerful mathematical framework based on simplicial complexes to uncover and quantify these multi-node interactions. However, its adoption has been limited by a lack of accessible software tools.

**Methods:**

We introduce a comprehensive Python package that implements the core methodology of Q-analysis. The package enables the construction of simplicial complexes from graphs or simplex lists and computes a suite of descriptive metrics, including structure vectors (FSV, SSV, TSV) and topological entropy. It features high-performance routines, integration with scikit-learn for machine learning workflows, and tools for statistical inference, such as permutation tests.

**Results:**

We demonstrate the package’s capabilities through a simulation study, revealing distinct higher-order topological signatures in scale-free versus configurational networks despite identical degree distributions. Application to the DBLP co-authorship dataset uncovered the evolution of collaborative structures over three decades, showing increased collaboration scale and shifts in higher-order connectivity patterns. Finally, in a network physiology application, the package identified significant disruptions in the higher-order organization of fMRI-derived brain networks in Major Depressive Disorder (MDD), characterized by a loss of high-dimensional functional components and increased fragmentation.

**Discussion:**

The developed package makes Q-analysis accessible to a broad research audience, facilitating the exploration of higher-order interactions in complex systems. The presented applications validate its utility across diverse domains, from social networks to neuroscience. By providing an open-source tool, this work bridges a gap in network science, enabling quantitative analysis of the intricate, multi-node structures that define real-world networks.

## 1 Introduction

Network analysis has become an indispensable tool in the contemporary data-driven world (Boccaletti et al., 2006; Zanin et al., 2016). It facilitates our comprehension of complex systems across diverse domains, from the intricate workings of various physiological systems (Berner et al., 2022; Günther et al., 2022), including the brain (Stam and Reijneveld, 2007; Yeo et al., 2011; Papo et al., 2014; Pisarchik and Hramov, 2023), to the analysis of social media connections (Zanin et al., 2012). As the volume of network data grows, the need for sophisticated methods to analyze the intricate connections and relationships within these network systems becomes increasingly critical.

Traditionally, complex network analysis has focused primarily on dyadic connections between pairs of nodes, represented by simple classical graphs (van der Vlag et al., 2022). This approach, commonly referred to as pairwise analysis, has provided important insights into the flow of information within networks and the formation of communities (Wang et al., 2010). In brain research, for example, it has elucidated communication pathways between different brain regions (Gross et al., 2021; Khorev et al., 2024) and has allowed the construction of a multigraph-based hierarchical model of consciousness impairment, where core large-scale functional network disruptions interact with state-dependent compensatory mechanisms (Kurkin et al., 2025).

However, scientists have recognized that studying solely pairwise connections often fails to capture the full complexity of real-world systems. Many such networks exhibit interactions that involve three or more components simultaneously, resulting in “group interactions” among the nodes of the network (Scagliarini et al., 2024; Yang et al., 2025). In social networks, for example, individuals often interact in groups rather than just in pairs. Similarly, in the brain, multiple regions often work together to perform specific tasks (Bishal et al., 2022; Scagliarini et al., 2024). Simple graphs lack the ability to adequately represent these group interactions, thereby limiting our understanding of complex network systems (Giusti et al., 2016).

Projecting multi-entity interactions onto a pairwise graph for analysis with traditional network methods leads to two fundamental problems. First is the loss of information: the original group nature of the interaction is irreversibly lost (Battiston et al., 2020). For example, a 3-clique (a triangle) in a graph could represent a single three-body interaction or three independent dyadic interactions; the pairwise representation makes these scenarios indistinguishable. Second is the ambiguity of higher-order structures. Consequently, any clique of size four or more in a pairwise graph has an ambiguous origin. Traditional edge-centric methods cannot adequately assess such structures, viewing them merely as dense clusters of pairwise links. To properly study them, a simplicial complex is first constructed from the graph (e.g., using the clique complex method), and Q-analysis is then applied. This two-step approach provides quantitative tools for investigating higher-order topology that are unavailable to standard pairwise methods.

This realization has spurred a growing interest in what is known as “higher-order” network analysis (Boccaletti et al., 2023). This approach attempts to capture and analyze these more complex interactions involving multiple nodes simultaneously. While higher-order analysis is not a novel concept, with roots going back several decades, its recent prominence is undeniable. This surge in popularity can be attributed to advancements in data collection methods, computational power, and analytical techniques. These developments have enabled the application of these methods to large-scale, real-world networks, including those that arise when solving physiological problems (Bick et al., 2021).

There are two primary approaches to representing and analyzing higher-order interactions in networks: hypergraphs and simplicial complexes. Hypergraphs extend the concept of classical graphs by allowing links, called “hyperedges”, to connect any number of nodes, not just pairs. This feature is particularly useful for modeling group interactions. For example, in a social network, a hyperedge could represent a group chat involving multiple participants (Boccaletti et al., 2023). In a brain physiological network, the hyperedges are the connected components in an absolute-valued brain functional connectivity network (Pisarchik and Hramov, 2023). Simplicial complexes, on the other hand, represent higher-order interactions as the geometric shapes. These shapes can be conceptualized as the higher-dimensional counterparts of nodes and edges. For example, a triangle in a simplicial complex could represent a three-way interaction. This approach originates from the field of algebraic topology (Sizemore et al., 2018). By using either hypergraphs or simplicial complexes, researchers can delve into the intricate interplay of multiple nodes within a network, providing deeper insights into the dynamics of complex systems.

While both hypergraphs and simplicial complexes provide frameworks for representing higher-order interactions in networks, they differ significantly in their structure and analytical capabilities. Hypergraphs, with their flexible representation of group interactions, provide an intuitive approach. However, they lack certain mathematical properties inherent to simplicial complexes. Simplicial complexes, by virtue of their geometric representation, allow for the exploration of network shape and structure in multiple dimensions. This enables the application of powerful mathematical tools, such as those from algebraic topology, to identify patterns that may elude traditional network analysis methods (Tadić et al., 2019). Given these distinct advantages, the remainder of this paper will focus exclusively on the analysis of higher-order interactions through the lens of simplicial complexes. This focus will leverage the inherent mathematical richness of this approach to uncover deeper insights into complex networks with different topologies, such as scale-free networks, random networks, and small-world networks.

A particularly insightful method for analyzing simplicial complexes is Q-analysis, originally proposed by R.H. Atkin in the 1970s (Atkin, 1974; Atkin, 1980). This method provides a systematic framework for examining the structure of simplicial complexes and quantifying their higher-order connectivity patterns. Despite its potential, Q-analysis has not gained widespread acceptance in network science, primarily due to the lack of readily available tools for its implementation.

At its core, Q-analysis offers a framework for understanding the multilevel structure of simplicial complexes. This approach introduces several key concepts:

• Q-connectivity: This basic concept defines two simplices as “q-near” if they share a q-dimensional face. This notion extends to “q-connectedness”, where simplices are considered q-connected if there exists a chain of simplices between them, with each pair in the chain being at least q-near. This property allows the grouping of simplices into q-connected components, revealing the structure of the complex at different dimensional levels.

• First structure vector (Q-vector): This central tool in Q-analysis is an integer vector where each entry, $Q_{q}$ , represents the number of q-connected components in the complex. The Q-vector provides a means to visualize the graded substructures of the simplicial complex at different connectivity levels.

• Additional structure vectors: Q-analysis also introduces other structure vectors, such as the second structure vector (counting simplices at each dimensional level) and the third structure vector (quantifying the degree of connectedness). These vectors provide different perspectives on the topology of the complex, allowing for a multifaceted analysis.

By applying these concepts, Q-analysis enables us to study the hierarchical, multilevel, and multidimensional organization of simplicial complexes. It reveals how simplices are connected at different dimensional levels, uncovering structural patterns that might not be apparent from a simpler analysis. In essence, Q-analysis provides a comprehensive approach to exploring the intricate structure of simplicial complexes, offering valuable insights into the topology and connectivity patterns of higher-order interactions in networks.

In social network analysis, Q-analysis has been used to study the structure of friendship networks (Freeman, 1980), the formation of scientific specialties (Hummon and Carley, 1993), and multiplexity in entrepreneurial networks (Bliemel et al., 2014). The method has proven particularly useful in revealing hidden multidimensional social structures (Maletić and Zhao, 2018) and modeling consensus formation in opinion dynamics (Maletić and Rajković, 2014). These applications demonstrate the power of Q-analysis in uncovering complex patterns of social interactions beyond simple dyadic relationships.

However, the potential of Q-analysis extends far beyond the social sciences. In neuroscience, for example, Q-analysis could provide valuable insights into the higher-order structure of brain networks (Kurkin et al., 2024; Andjelk et al., 2020). While current research in this area often relies on graph theory and pairwise connectivity measures (Wang et al., 2021; Motlaghian et al., 2022), Q-analysis provides a framework for capturing more complex, multidimensional interactions between brain regions. This approach may be particularly relevant given the growing recognition of the importance of higher-order connectivity in brain function (Giusti et al., 2016; Hindriks et al., 2024). There are a few pioneering studies that have applied Q-analysis in neuroscience, such as the investigation of brain-to-brain coordination networks (Tadić et al., 2019) and the analysis of human brain networks through order statistics (Das et al., 2023). These rare examples demonstrate the potential of Q-analysis to reveal novel insights into brain structure and function, but also highlight the current underutilization of this method in the field.

In other fields where complex systems exhibit higher-order interactions, the potential of Q-analysis remains largely unexplored. For example, in systems biology, physiology, ecology, or climate science, where intricate relationships between multiple components are common, Q-analysis could provide a novel perspective on system dynamics and structure. The ability of the method to quantify connectivity at different dimensional levels makes it well suited to analyzing hierarchical and multi-scale phenomena in these fields.

A notable exception outside the social sciences is the application of Q-analysis to evaluate the performance of distribution systems (Duckstein, 1983). This study demonstrated how Q-analysis can be used to identify potential bottlenecks and improve operational characteristics in complex networks, suggesting its potential utility in areas such as logistics, supply chain management, and infrastructure planning.

The limited adoption of Q-analysis beyond the social sciences can be attributed to several factors, including the lack of readily available computational tools and the perceived complexity of the method compared to traditional network analysis techniques. However, with the burgeoning interdisciplinary research landscape and the growing demand for tools to analyze higher-order interactions, Q-analysis represents a promising avenue for researchers in different scientific fields to gain deeper insights into complex networks. Q-analysis is an exploratory framework for analyzing higher-order network structures. Its primary outputs are a set of descriptive metrics, such as structure vectors and topological entropy, which serve as quantitative summaries of network topology across different dimensional levels. These parameters characterize the underlying organization of a complex system, providing a basis for comparison across networks and for generating new hypotheses about their structure.

Despite its conceptual utility, the practical application of Q-analysis has been infrequent, partly due to a lack of available and maintained software. The contribution of this work is to help address this gap by providing q-analysis, a Python package that implements the core methodology. The package is intended to make the tools of Q-analysis more accessible, thereby facilitating the study of higher-order network structures within the scientific Python ecosystem.

The structure of the paper is as follows. Section 2.1 lays out the mathematical framework of Q-analysis, including the concepts of simplicial complexes and Q-analysis metrics. Section 2.2 introduces the developed Python package, detailing its core functionality and its integration into the wider scientific computing ecosystem. Section 3.1 showcases the package’s capabilities through a simulation study, comparing Q-analysis metrics across different network types. Section 3.2 demonstrates the package’s utility on a real-world co-authorship dataset, analyzing its structural evolution over time. Finally, Section 3.3 provides an example of using the package to study disruptions in the fMRI-derived brain network caused by major depressive disorder.

## 2 Materials and methods

### 2.1 Mathematical background

In this section we will briefly lay out the basic definitions of simplicial topology and Q-analysis. In Section 2.1.1 we will explain what simplices are. In Section 2.1.2 we will discuss the main terms of Q-analysis. Section 2.1.3 introduces the concept of graded parameter sets. Sections 2.1.4, 2.1.5 formally define structure vectors and other Q-analysis metrics.

#### 2.1.1 Simplicial complexes

A simplicial complex serves as the foundational mathematical structure for Q-analysis (Atkin, 1980). Unlike traditional network analysis, which focuses on dyadic (pairwise) relationships, simplicial complexes capture multi-node interactions and hierarchical structures.

Let V be a finite set whose elements are called vertices. A *p-simplex* σ is an ordered subset of V containing p+1 vertices, written as $\sigma =\left\langle v_{0},v_{1},\ldots ,v_{p}\right\rangle$ (Johnson, 2014). The dimension of simplex σ , denoted as dim(σ) , is defined as:

$$\text{dim}\left(\sigma \right)=|\sigma |-1=p,$$

where |σ| represents the number of vertices in σ .

A simplicial complex K is a collection of simplices satisfying the property that if σ∈K and $\sigma^{\prime }\subset \sigma$ (i.e., $\sigma^{\prime }$ is a face of σ ), then $\sigma^{\prime }\in K$ (Johnson, 2014). In other words, a simplicial complex includes all faces of its simplices.

A simplex $\sigma^{\prime }$ is a *face* of simplex σ if $\sigma^{\prime }\subset \sigma$ (Johnson, 2014). We denote this relationship as $\sigma^{\prime }⪯\sigma$ . The dimension of a face $\sigma^{\prime }$ is less than or equal to the dimension of σ .

A simplicial complex with m simplices and n vertices can be represented by an *incidence matrix* Λ which is an m×n matrix where:

$$\Lambda_{ij}=\left\{\begin{array}{cc} 1,\; & \text{if vertex }j\text{ belongs to simplex }i,\\ 0,\; & \text{otherwise}. \end{array}\right.$$

**FIGURE 1:**
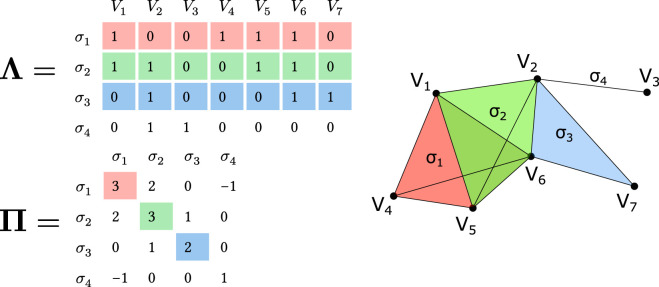
Matrix representation of a simplicial complex and q-nearness visualization. Top: Incidence matrix Λ showing relationships between simplices ( $\sigma_{1}$ - $\sigma_{4}$ ) and vertices ( $V_{1}$ - $V_{7}$ ). Each row represents a simplex, with 1’s indicating included vertices. Bottom: Connectivity matrix $\Pi =\Lambda \cdot \Lambda^{T}-\Omega$ with diagonal elements showing simplex dimensions (e.g., $\Pi_{11}=3$ means $\sigma_{1}$ is a 3-simplex) and off-diagonal elements showing shared face dimensions (e.g., $\Pi_{12}=2$ indicates $\sigma_{1}$ and $\sigma_{2}$ share a 2-dimensional face). Right: Geometric representation of the simplicial complex with colored regions showing higher-dimensional simplices. Note that $\sigma_{1}$ and $\sigma_{3}$ are 0-near (sharing only $V_{6}$ ), while $\sigma_{1}$ and $\sigma_{2}$ are 2-near (sharing face $\left\{V_{1},V_{5},V_{6}\right\}$ ).

Thus, the incidence matrix Λ is a binary matrix that encodes the relationship between simplices and vertices, with each row corresponding to a simplex and each column corresponding to a vertex. An example of such a matrix is shown in Figure 1 (top). Using the incidence matrix, we can compute the *connectivity matrix* Π which encodes the q-nearness between simplices, similar to how the adjacency matrix of a graph encodes the adjacency between nodes.

$$\Pi =\Lambda \cdot \Lambda^{T}-\Omega ,$$ (1)

where Ω is an all-ones matrix. The Ω term is used to adjust the face dimension calculation, as q-nearness is defined by the dimension of shared faces (number of shared vertices minus one). Without this subtraction, the matrix product would give the number of shared vertices rather than the dimension of the shared face. Figure 1 (bottom) shows an example of a connectivity matrix.

#### 2.1.2 Q-analysis fundamentals

Two simplices σ and $\sigma^{\prime }$ are *q-near* if they share a common face of dimension at least q , meaning there exists a simplex τ such that:

$$\tau ⪯\sigma \text{ and }\tau ⪯\sigma^{\prime }\text{ with }\text{dim}\left(\tau \right)\geq q.$$

Here, τ⪯σ indicates that τ is a face of σ . This concept is illustrated in Figure 1, where simplices $\sigma_{1}$ and $\sigma_{2}$ are 2-near as they share a 2-dimensional face, while $\sigma_{1}$ and $\sigma_{3}$ are only 0-near as they share just one vertex $\left(V_{6}\right)$ . In the connectivity matrix Π , the q-nearness between two simplices can be directly read from the corresponding entry: $\Pi_{ij}\geq q$ indicates that simplices i and j are q-near.

Extending this concept, simplices σ and $\sigma^{\prime }$ are *q-connected* if there exists a sequence of simplices $\sigma_{1},\sigma_{2},\ldots ,\sigma_{n}$ with $\sigma_{1}=\sigma$ , $\sigma_{n}=\sigma^{\prime }$ , and $\sigma_{i}$ is q-near to $\sigma_{i+1}$ for i=1,2,…,n−1 . This sequence forms a chain of q-connection between σ and $\sigma^{\prime }$ .

Thus we can define a *q-connected component* as a maximal q-connected subset of simplices. A single simplex of order q can form a q-connected component by itself if it is not q-near to any other simplex. For example, in Figure 1, simplices $\sigma_{1}$ , $\sigma_{2}$ , and $\sigma_{3}$ form a 1-connected component because $\sigma_{1}$ and $\sigma_{2}$ share a 2-dimensional face (which makes them at least 1-near), and $\sigma_{2}$ and $\sigma_{3}$ share a 1-dimensional face. This creates a chain of 1-nearness from $\sigma_{1}$ to $\sigma_{3}$ through $\sigma_{2}$ . For q=2 , there are two 2-connected components: one containing $\sigma_{1}$ and $\sigma_{2}$ (which share a 2-dimensional face) and another containing only $\sigma_{3}$ (which is itself a 2-simplex but does not share any 2-dimensional face with other simplices). The parameter q thus acts as a resolution parameter: lower values of q impose a weaker connectivity condition, resulting in fewer, larger components, while higher values of q impose a stricter condition, leading to a more fragmented structure.

#### 2.1.3 Graded parameter sets

Q-analysis employs a variety of metrics to quantify the structural and topological properties of simplicial complexes. In this paper, we focus on a specific category of metrics that we refer to as *graded parameter sets*.

A *graded parameter set*, a notion introduced by Atkin (1980), is a collection of values graded by the dimensional level q of the simplicial complex, where q acts as the independent variable. This category includes the structure vectors, which will be defined in the following section. Other metrics, such as topological entropy, also fall into this category. These graded parameter sets provide a dimensional perspective on the complex’s topology, complementing other metrics in Q-analysis that will be introduced in subsequent sections.

#### 2.1.4 Q-analysis structure vectors

Q-analysis employs various structure vectors to quantify the connectivity patterns and topological features of simplicial complexes.

**FIGURE 2:**
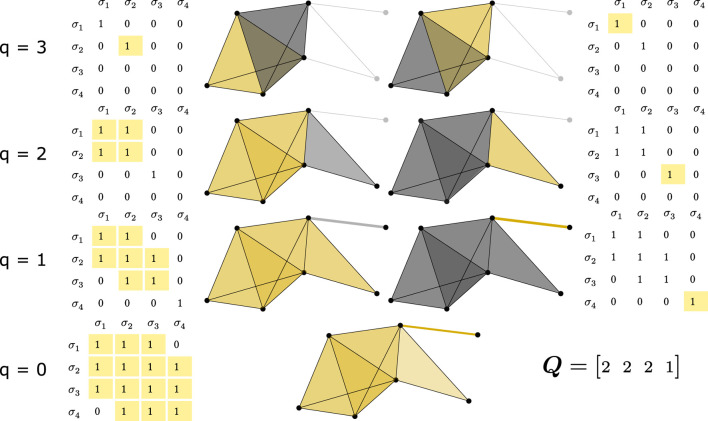
Q-analysis process for the FSV, illustrated through the q-connected components identification. This figure demonstrates the analysis of the simplicial complex from Figure 1. Each column (with its thresholded matrix and graphical representation) represents a separate connected component. Rows show connected components of order q, starting from the highest q (3 in this case). Simplices comprising a connected component are colored yellow, while others of the same order are grey. Thresholding refers to comparing values of connectivity matrix Π with corresponding q value—entries greater than or equal to q are replaced with 1, otherwise 0. By thresholding Π from Figure 1 at each q and applying a connected component search algorithm (interpreting the thresholded matrix as an adjacency matrix), we find: for q = 3, two components; for q = 2, two components; for q = 1, two components; and for q = 0, one component. These counts form the First Structure Vector Q=[2; 2; 2; 1] .

The *First Structure Vector* (FSV), denoted as Q , captures the number of q-connected components for each dimensional level q . For a simplicial complex of dimension d , the FSV is

$$Q=\left[Q_{d},Q_{d-1},\ldots ,Q_{1},Q_{0}\right],$$

where $Q_{q}$ represents the number of distinct q-connected components at dimension q (Atkin, 1980). In other words, $Q_{q}$ is the number of q-connected components in the simplicial complex. We can understand $Q_{q}$ conceptually as counting the number of groups of simplices that are connected through chains of q-nearness relations. This is analogous to finding connected components in a graph, but instead of using adjacency, we use the q-nearness relation (where two simplices are connected if they share a face of dimension at least q). This relation is captured in the connectivity matrix Π from Equation 1, where an entry $\Pi_{ij}\geq q$ indicates that simplices i and j are q-near. Figure 2 illustrates this component identification process for different values of q .

The *Second Structure Vector* (SSV), denoted as n , counts the number of simplices at each dimensional level:

$$n=\left[n_{d},n_{d-1},\ldots ,n_{1},n_{0}\right],$$

where $n_{q}$ is the count of simplices with dimension greater than or equal to q (Atkin, 1980). Formally, for a simplicial complex K :

$$n_{q}=|\left\{\sigma \in K:\text{dim}\left(\sigma \right)\geq q\right\}|,$$

where |⋅| denotes the cardinality of the set.

The use of a cumulative count (≥q) makes the SSV monotonically non-increasing, which is a prerequisite for its use in normalizing the Third Structure Vector.

The Third Structure Vector (TSV), denoted as $\bar{Q}$ , quantifies the degree of connectedness at each dimensional level:

$${\bar{Q}}_{q}=1-\frac{Q_{q}}{n_{q}}.$$

TSV provides a normalized measure of connectivity, with values closer to one indicating higher connectivity. This is because if there are fewer q-connected components than simplices, it means that the simplices are more connected. Mathematically, when $Q_{q}\ll n_{q}$ , then $\frac{Q_{q}}{n_{q}}\approx 0$ , resulting in ${\bar{Q}}_{q}\approx 1$ (high connectivity). Conversely, if the number of q-connected components is equal to the number of simplices (i.e., $Q_{q}=n_{q}$ ), then ${\bar{Q}}_{q}=1-n_{q}/n_{q}=0$ , indicating that the simplices are not connected at all (each simplex is a separate q-connected component).

For a concrete example, consider a system represented by two 2-simplices, $\sigma_{1}=\left\langle v_{1},v_{2},v_{3}\right\rangle$ and $\sigma_{2}=\left\langle v_{2},v_{3},v_{4}\right\rangle$ . The maximum dimension is d=2 . The structure vectors are computed for this set of two simplices as follows:

• Second Structure Vector (n) : For any q∈{0,1,2} , both simplices have dimension ≥q . Thus, $n_{2}=2$ , $n_{1}=2$ , and $n_{0}=2$ , giving n=[2,2,2] .

• First Structure Vector (Q) :

• For q=2 , the simplices are not 2-near, as their shared face $\left\langle v_{2},v_{3}\right\rangle$ has dimension 1. They form two separate 2-connected components, so $Q_{2}=2$ .

• For q=1 , the simplices are 1-near because their shared face has dimension 1≥1 . They form a single 1-connected component, so $Q_{1}=1$ .

• For q=0 , they are also 0-near, forming one component, so $Q_{0}=1$ .
  This results in the vector Q=[2,1,1] .

• Third Structure Vector $\left(\bar{Q}\right)$ : Using ${\bar{Q}}_{q}=1-Q_{q}/n_{q}$ :

• ${\bar{Q}}_{2}=1-2/2=0$ .

• ${\bar{Q}}_{1}=1-1/2=0.5$ .

• ${\bar{Q}}_{0}=1-1/2=0.5$ .

This gives the vector $\bar{Q}=\left[0,0.5,0.5\right]$ .

#### 2.1.5 Other Q-analysis metrics

Q-analysis methodology has been extended with additional metrics by subsequent researchers. One such metric is *topological entropy*, introduced by Andjelkovic et al. (Andjelković et al., 2015), which quantifies the diversity of simplicial participation at each dimensional level:

$$S_{Q}\left(q\right)=-\underset{i}{\sum }\frac{p_{i}^{q}\log p_{i}^{q}}{\log M_{q}},$$

where

$$p_{i}^{q}=\frac{Q_{i}^{q}}{\sum_{i}Q_{i}^{q}}$$

represents the normalized participation of vertex i in q-dimensional simplices, $Q_{i}^{q}$ is the number of q-dimensional simplices containing vertex i , $M_{q}=\sum_{i}\left(1-\delta_{Q_{i}^{q},0}\right)$ counts vertices with non-zero participation at level q , and $\delta_{Q_{i}^{q},0}$ is the Kronecker delta function. Due to its definition, this measure can be considered a graded parameter set.

Topological entropy provides an information-theoretic perspective on the distribution of vertex participation in simplices of dimension q . It measures how evenly vertices participate in the higher-order structures of the network. High q-topological entropy value (close to 1) indicate that vertices participate relatively evenly in q-dimensional simplices. This suggests a decentralized structure where interactions are well-distributed across the network, with no single vertex dominating the higher-order connectivity. On the other hand, low q-topological entropy values (close to 0) indicate that participation in q-dimensional simplices is concentrated among a few vertices. This suggests a more centralized structure with hub-like vertices that dominate the higher-order interactions.

The *eccentricity* of a simplex σ with respect to another simplex $\sigma^{\prime }$ quantifies their topological distance (Johnson, 2014):

$$\text{ecc}\left(\sigma |\sigma^{\prime }\right)=\frac{\left|\sigma \backslash \sigma^{\prime }\right|}{\left|\sigma \right|}=\frac{\text{number of vertices in }\sigma \text{ not shared with }\sigma^{\prime }}{\text{number of vertices in }\sigma }$$

The *family eccentricity* of a simplex σ with respect to a family of simplices F is (Johnson, 2014):

$$\text{ecc}\left(\sigma |F\right)=\min\left\{\text{ecc}\left(\sigma |\sigma^{\prime }\right)|\sigma^{\prime }\in F\right\}$$

The *topological dimensionality* is another important metric in Q-analysis introduced by Andjelković et al. (2015) that characterizes the participation of vertices in the simplicial complex. For a vertex v∈V , its topological dimensionality d(v) is formally defined as:

$$D\left(v\right)=\overset{d}{\underset{q=0}{\sum }}Q_{v}^{q}$$

where $Q_{v}^{q}$ represents the number of q -dimensional simplices containing vertex v , and d is the highest simplex dimension in the complex.

The topological dimensionality can be expressed in terms of the incidence matrix Λ of the simplicial complex. Specifically, for all vertices, the topological dimensionality vector d is given by:

$$d=\text{diag}\left(\Lambda^{T}\cdot \Lambda \right),$$

where diag(⋅) extracts the diagonal elements of a matrix. This metric extends the concept of vertex degree from graph theory to the higher-dimensional setting of simplicial complexes. While the degree of a vertex in a graph counts only its pairwise connections, topological dimensionality accounts for participation in multi-node interactions across all dimensions. Vertices with high topological dimensionality function as hubs in the complex, participating in numerous higher-order structures.

### 2.2 Package description

The q-analysis package provides a Python implementation of tools for simplicial complex analysis, designed for studying higher-order interactions in complex systems. It offers data structures for representing simplicial complexes, methods for computing Q-analysis metrics, and tools for integrating these metrics into machine learning and statistical analysis workflows.

#### 2.2.1 Core components

The central class in the package is SimplicialComplex, which represents a simplicial complex as a collection of simplices. A complex can be instantiated in two primary ways: directly from a list of simplices, or from a graph’s adjacency matrix using the from_adjacency_matrix () static method. The latter approach identifies the maximal cliques of the graph, treating each as a simplex. This allows for the application of Q-analysis to traditional graph structures.

The core methods of the SimplicialComplex class are summarized in Table 1.

**TABLE 1:** Core methods of the SimplicialComplex class.

Method	Description
__init__(simplices)	Constructs a complex from a list of simplices. Each simplex is a list or set of vertex indices
from_adjacency_matrix (adj)	Creates a complex from a graph’s adjacency matrix by finding all maximal cliques
graded_parameters (desired_vectors)	Computes specified Q-analysis vectors (e.g., FSV, SSV) across all relevant dimensions and returns them in a GradedParameters container
q_connected_components(q)	Returns the number of q-connected components for a specific level q
q_connected_components_labeled(q)	Returns the q-connected components, with each simplex labeled by its component ID.
topological_dimensionality ()	Computes the number of simplices each vertex belongs to, returned as a NodeParameterSet.
eccentricity (simplex_a, simplex_b)	Computes the eccentricity of one simplex relative to another
family_eccentricity (simplex,...)	Computes the minimum eccentricity of a simplex relative to a family of other simplices

The package uses several data container classes to organize results. Methods like graded_parameters () return a GradedParameters object, which holds multiple GradedParameterSet instances–one for each computed vector (e.g., FSV, SSV). This container provides methods to access individual vector sets. Similarly, topological_dimensionality () returns a NodeParameterSet, which stores per-vertex values. All these container objects provide a to_dataframe () method to export the data into a pandas DataFrame in a tidy format, suitable for analysis and visualization.

##### 2.2.1.1 Object instantiation

A SimplicialComplex is created by passing a list of simplices to its constructor.

>>> SimplicialComplex(simplices)

Where simplices is a list of lists/sets, e.g., [[0, 1, 2], [1, 2, 3]].

Alternatively, it can be created from a NumPy adjacency matrix. The method uses networkx to find cliques.

SimplicialComplex.from_adjacency_matrix(adj_matrix)

##### 2.2.1.2 Computing Q-analysis vectors

The primary method for computing graded parameters is graded_parameters ().

>>> graded_parameters(desired_vectors=None)

The desired_vectors argument is a list of strings specifying which vectors to compute (e.g., [’FSV’, ’SSV’, ’TSV’]). By default, it computes all available vectors. The method returns a GradedParameters object, which bundles the results into GradedParameterSet objects for each vector. This structure facilitates easy access and conversion to pandas DataFrames for further analysis.

##### 2.2.1.3 Connected components

The number of q-connected components is a key metric for the First Structure Vector (FSV). While the Python method for retrieving this count takes a specific integer q , the underlying calculation is optimized.

q_connected_components(q)

Upon first call, the package computes the components for all possible q-levels at once using a backend implemented in Rust for efficiency. This approach operates directly on the list of simplices and caches the results. Subsequent calls for different q values access the cached data without re-computation.

For a more detailed analysis that identifies which component each simplex belongs to, the following method can be used:

q_connected_components_labeled(q)

This method returns a list of sets, where each set contains the indices of simplices belonging to one component.

##### 2.2.1.4 Vertex-level metrics

The package computes vertex-level metrics such as topological dimensionality and eccentricity.

The topological_dimensionality () method calculates the number of simplices that each vertex is a part of. The result is returned as a NodeParameterSet object. If node_names are provided, they are used to label the vertices in the output.

topological_dimensionality(node_names=None)

The eccentricity of a simplex with respect to another can be computed using the eccentricity method.

eccentricity(simplex_a, simplex_b)

Where simplex_a and simplex_b can be specified as either integer indices or sets of vertices. The method calculates the proportion of vertices in simplex_a that are not in simplex_b.

For broader comparisons, the family_eccentricity method computes the minimum eccentricity of a given simplex relative to an entire family of other simplices in the complex.

family_eccentricity(simplex)

By default, it compares the simplex against all other simplices in the complex.

#### 2.2.2 Integration with scikit-learn

The package includes several classes that follow the scikit-learn transformer API, allowing for the integration of Q-analysis into machine learning pipelines (similar to the approach in giotto-tda). These transformers operate on collections of graphs or simplicial complexes. The main transformers are listed in Table 2.

**TABLE 2:** Q-analysis transformers for use with scikit-learn.

Class	Description
GradedParametersTransformer	Computes Q-analysis structure vectors for a collection of graphs. Takes an iterable of adjacency matrices, converts each to a simplicial complex via maximal cliques, and returns their corresponding structure vectors
QConnectedComponents	Computes q-connected component labels for a collection of simplicial complexes. Takes an iterable of simplex lists
GraphCliqueFilter	A transformer that filters a graph by keeping only edges that belong to cliques of at least a specified dimension q . It internally builds a simplicial complex and uses the SimplexProjection transformer
SimplexProjection	Projects a simplicial complex to a graph representation. Edges connect vertices that co-occur in simplices. Edges can be weighted by the number of co-occurrences

For example, the GradedParametersTransformer can be used to extract features from a set of networks for a subsequent classification or regression task.

#### 2.2.3 Downstream integration for statistical inference

The package provides functions to format Q-analysis outputs for use with standard statistical libraries, bridging descriptive metrics and formal inference. These helper functions, located in the q_analysis.utils and q_analysis.stat modules, facilitate the use of outputs with libraries like scipy.stats.

##### 2.2.3.1 Permutation testing

The package supports permutation testing by providing data preparation utilities. Functions such as pad_structure_vectors () and adj_matrices_to_q_analysis_vectors () from the q_analysis.utils and q_analysis.stat modules generate uniformly-sized NumPy arrays of Q-analysis vectors from networks of varying sizes. These arrays can be used directly with functions like scipy.stats.permutation_test to assess the statistical significance of differences between two groups of networks.

##### 2.2.3.2 Consensus network-based permutation test

For group-level comparisons, the consensus_statistic function offers a consensus-based testing approach. This method first constructs a single consensus network for each group (Pisarchik et al., 2023), representing the shared topological structure. Q-analysis vectors are then computed for these consensus networks, and the test statistic is then estimated. The null distribution is generated by permuting group labels, re-computing consensus networks, and calculating the statistic for each permutation. This is useful for identifying systematic structural differences between populations of networks.

#### 2.2.4 Implementation and performance

Performance-critical routines are compiled methods written in Rust with memory preallocation logic, which are accessed from Python. The algorithm for finding q-connected components operates directly on the list of simplices. The time complexity of this hierarchical algorithm is sensitive to the complex’s structure; in the worst case, it approaches $O\left(d_{\max}\cdot s_{\max}\cdot m^{2}\right)$ , where $d_{\max}$ is the maximum dimension of the complex, $s_{\max}$ is the maximum simplex size, and m is the number of simplices. The memory footprint scales primarily with the total number of vertices across all simplices, $O\left(\sum_{i=1}^{m}|s_{i}|\right)$ , and the storage of the resulting components. Computational limits are a consideration for large simplicial complexes; on standard consumer hardware (e.g., a laptop with 16 GB of RAM), the package can handle complexes with up to several hundred thousand simplices, though the exact limit depends on their average size and connectivity.

#### 2.2.5 Input validation and robustness

The package provides basic input robustness. Degenerate simplices containing repeated vertices are handled by casting each simplex to a ‘set’ during SimplicialComplex instantiation, which implicitly removes duplicates. When creating a complex from an adjacency matrix, input validation is delegated to the networkx library.

#### 2.2.6 Computational details

The results in this paper were obtained using Python 3.10 with numpy 1.23.5 (Harris et al., 2020), scipy 1.10.1 (Virtanen et al., 2020), networkx 3.1, statannotations 0.7.1 (Charlier et al., 2023) and matplotlib 3.7.1 (Virtanen et al., 2020) packages.

## 3 Results

### 3.1 A showcase of the package on a simulation study: scale-free networks *versus* configurational networks with the same degree distribution

In this section, we showcase the package’s capabilities through a simulation study comparing Q-analysis metrics across different network types. We compare scale-free networks with configurational networks with same degree distribution. We start by generating a set of scale-free networks with fixed amount of nodes N=50 . We use the Barabási-Albert model (Albert and Barabási, 2002) to generate the scale-free networks, with M=10 . For each scale-free network, we generate a configurational network with same degree distribution. A configurational networks is generated using Havel-Hakimi algorithm (Havel, 1955; Hakimi, 1962). We generate 100 scale-free networks and 100 configurational networks. This data can be generated using the following code:

>>> from q_analysis.examples.scale_free_configurational import generate_networks
>>> import numpy as np
>>> N_SAMPLES, N_NODES, M_PARAMETER = 100, 100, 8
>>> scale_free_networks, configurational_networks = generate_networks(
…   N_NODES,
…   M_PARAMETER,
…   N_SAMPLES,
… )
>>> networks = np.concatenate([scale_free_networks, configurational_networks])

Method generate_networks uses seeded generation for reproducibility. Resulting variables scale_free_networks and configurational_networks are lists of adjacency matrices in the form of numpy arrays.

We may then compute Q-analysis metrics for both network types. There are several ways to do this using the package. In this example, we will use the most straightforward approach by first building a simplicial complex from the adjacency matrix and then computing q-analysis metrics from the simplicial complexes.

>>> from q_analysis.simplicial_complex import SimplicialComplex
>>> from itertools import product
>>> index = product([’Scale free’, ’Configurational’], range(N_SAMPLES))
>>> simplicial_complex_metrics = [
…   SimplicialComplex
…     .from_adjacency_matrix(network)
…     .graded_parameters()
…     .to_dataframe()
…     .assign(Network=net_type, Sample=sample_id)
…   for network, (net_type, sample_id) in zip(networks, index)
… ]

We can then concatenate those datasets and visualize them:

>>> import pandas as pd
>>> from matplotlib import pyplot as plt
>>> from q_analysis.viz import plot_q_analysis_vectors
>>> structure_vectors_df = pd.concat(
simplicial_complex_metrics,
ignore_index=True
)
>>> plot_q_analysis_vectors(
…   structure_vectors_df,
…   hue=“Network”,
…   height=3,
…   col_wrap=2,
…   legend_out=False
… )
>>> plt.show()

**FIGURE 3:**
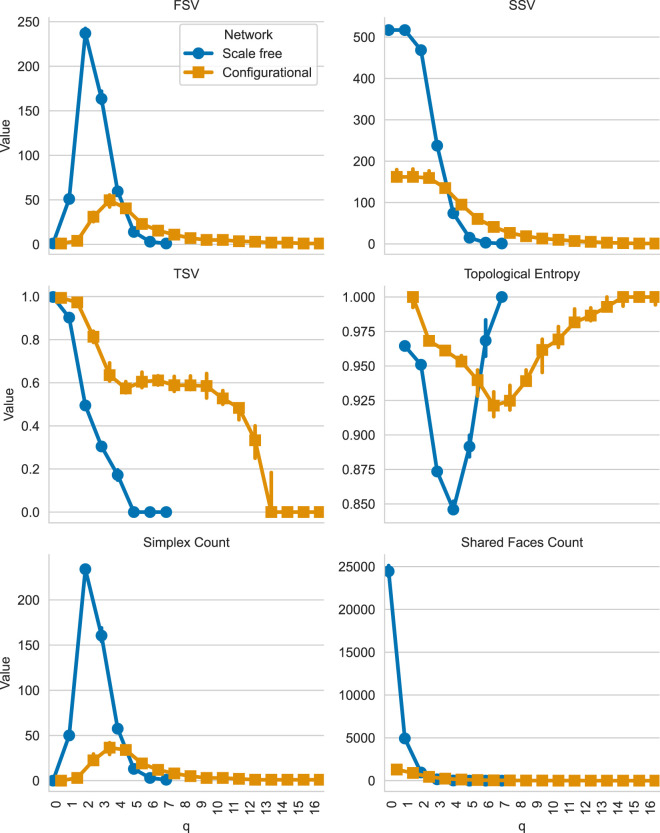
Comparison of Q-analysis median structure vectors across scale-free and configurational networks with identical degree distributions. Blue markers represent scale-free networks, while orange markers represent configurational networks. The plot shows the FSV, SSV, TSV, topological entropy, number of simplices, and number of shared faces ( y -axis) at each q value ( x -axis).

**FIGURE 4:**
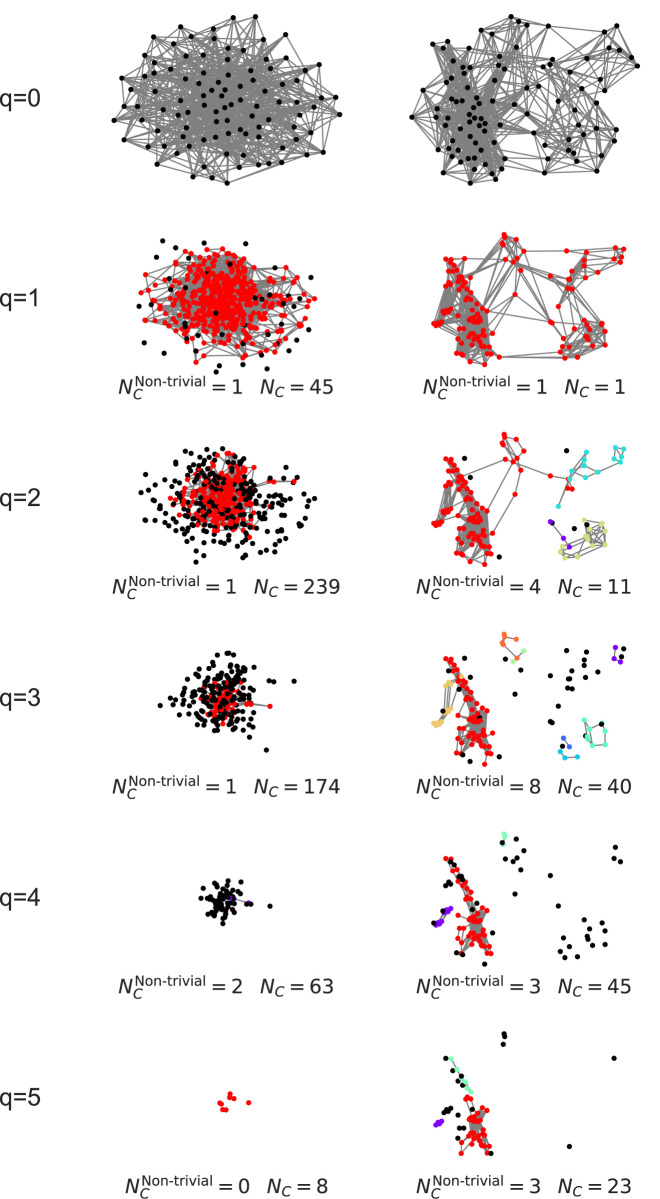
Comparison of Q-analysis connected components decomposition for scale-free (left) and configurational (right) networks. Each row represents graph of simplices of order q and higher, with coordinates calculated as mean of simplex’s node coordinates. Original graphs are plotted using NetworkX’s generated spring layout. Colored nodes represent simplices involved in a component with more than one simplex, black nodes represent simplices involved in 1-simplex component. ${\text{N}}_{\text{C}}$ is the overall number of components, ${\text{N}}_{\text{C}}^{\text{Non}-\text{trivial}}$ is the number of components with more than one simplex.

**FIGURE 5:**
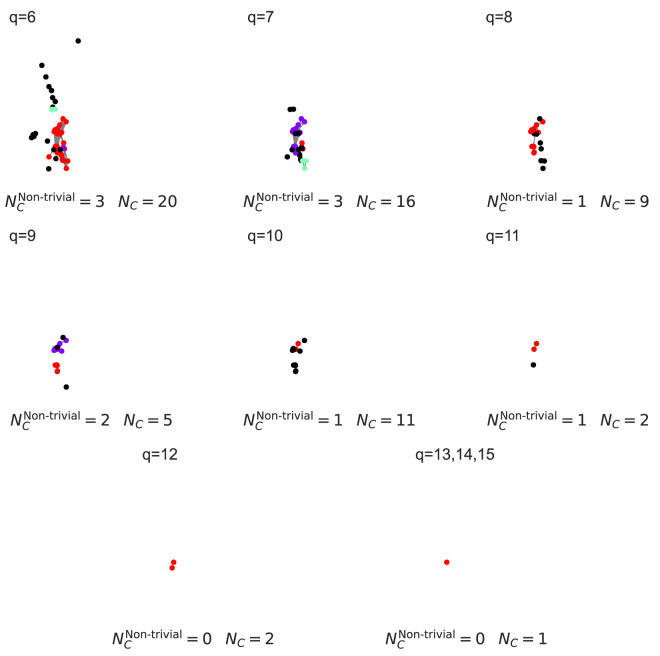
Decomposition of higher-order components for the configurational network for q>5 . The corresponding scale-free network has no components at these levels.

This will give us the plot shown in Figure 3. The results show that scale-free networks concentrate connectivity in lower-dimensional structures with pronounced drop-offs at higher orders, whereas configurational networks distribute connectivity more evenly across dimensions. TSV shows that configurational networks maintain higher connectivity at intermediate and higher q values, suggesting less fragmentation in their higher-order structures. Similarly, topological entropy measurements indicate more uniform vertex participation in higher-order structures in configurational networks compared to scale-free networks, which display noticeable entropy dips at middle q values. These topological differences can be visually understood by examining Figures 4, 5, which illustrates how connected components evolve across different q values for both network types. This visual representation reveals how the organizational principles governing these networks—preferential attachment in scale-free networks *versus* the degree-preserving reconfiguration in configurational networks—produce fundamentally different higher-order structures despite identical degree sequences.

There is also a possibility to compute consensus networks and their corresponding q-analysis metrics. For the case of modeled networks this is of limited value—since the nodes do not bear any semantic meaning and are not shared between generated networks. However, this process can be useful for real networks, where different networks have the same nodes, e.g., different brain regions in different subjects. Such analysis is presented in work of Kurkin et al. (2024). The consensus-based permutation test and its results visualization can be computed using the code below. We can start by computing consensus networks:

>>> from q_analysis.stat import calculate_consensus_adjacency_matrix
>>> from q_analysis.transformers import GradedParametersTransformer
>>> consensus_scale_free_vectors, consensus_configurational_vectors = (
…    GradedParametersTransformer().fit_transform(
…      [
…       calculate_consensus_adjacency_matrix(scale_free_networks),
…       calculate_consensus_adjacency_matrix
        (configurational_networks),
…      ]
…    )
… )

Then we can compute the permutation test. We use the consensus_statistic () function to compute the test statistic. We compute maximum order of consensus simplicial complexes beforehand so that computed statistics arrays have the same size. We use the scipy’s permutation_test () function to compute the p-values.

>>> from scipy import stats
>>> from q_analysis.stat import consensus_statistic
>>> max_order = len(consensus_scale_free_vectors)
>>> stats_res = stats.permutation_test(
…  [scale_free_networks, configurational_networks],
…  statistic=lambda a, b, axis: consensus_statistic(
…   a, b, max_order=max_order, edge_inclusion_threshold=0.95
…  ),
…  n_resamples=10000,
…  vectorized=True,
…  batch=100,
…  axis=1,
… )

Then we make a dataframe from the p -values and visualize the results, what can be done using the same method as in the previous example:

>>> from q_analysis.simplicial_complex import GradedParameters
>>> p_values_df = GradedParameters.from_numpy
    (stats_res.pvalue).to_dataframe()
>>> consensus_vectors_df = pd.concat([
…   GradedParameters.from_numpy (consensus_vector)
…   .to_dataframe()
…   .assign(Network=network)
…   for network, consensus_vector in zip(
…     [’Scale free’, ’Configurational’],
…     [consensus_scale_free_vectors,
consensus_configurational_vectors]
…   )
… ], ignore_index=True)
>>> plot_q_analysis_vectors(
…    consensus_vectors_df,
…    pvalues_df=p_values_df,
…    hue=’Network’,
…    height=3,
…    col_wrap=2,
…    legend_out=False
… )
>>> plt.show()

**FIGURE 6:**
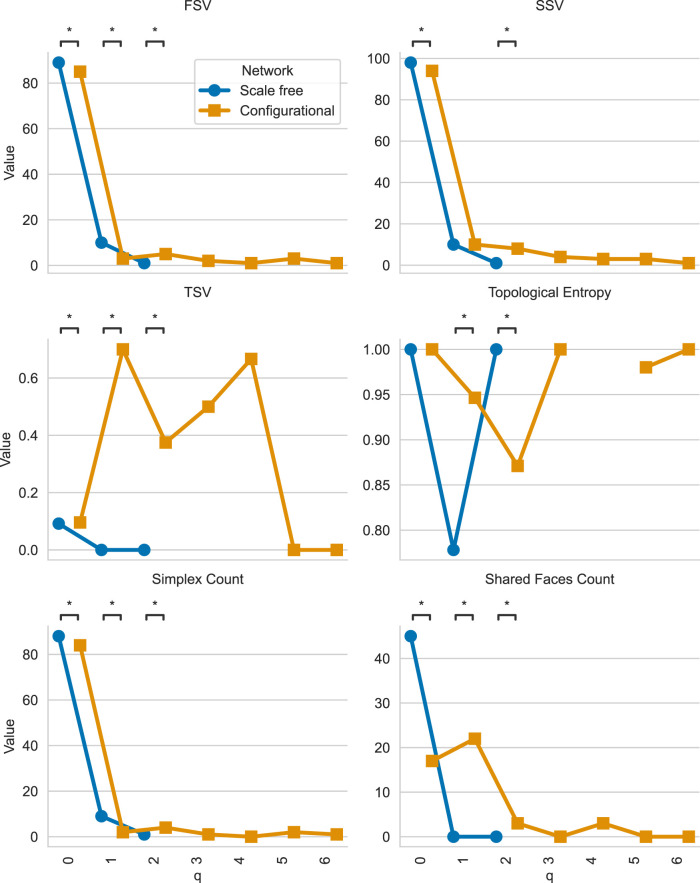
Comparison of Q-analysis consensus structure vectors across scale-free and configurational networks with identical degree distributions. Blue markers represent scale-free consensus network q-analysis metrics, while orange markers represent configurational consensus network q-analysis metrics. The plot shows the FSV, SSV, TSV, topological entropy, number of simplices, and number of shared faces ( y -axis) at each q value ( x -axis). P-values are computed using the consensus network-based permutation test and significant comparisons are marked with ‘*’.

The resulting plot is shown in Figure 6. We can see that the consensus networks differ from the individual networks by having less higher-order structures. This is expected since the consensus networks have less edges and thus less higher-order structures.

It is also possible to get the plot for topological dimensionality:

>>> import seaborn as sns
>>> topological_dimensionality_df = pd.concat([
…    SimplicialComplex
…     .from_adjacency_matrix(network)
…     .topological_dimensionality()
…     .to_dataframe()
…     .assign(Network=net_type)
…     .set_index([’Network’,’Node’])
…    for network, net_type in zip(
…      [scale_free_networks[0],configurational_networks[0]],
…      [’Scale free’,’Configurational’]
…    )
… ])
>>> sns.barplot(
…   data=topological_dimensionality_df,
…   x=“Node”,
…   y=“Topological Dimensionality”,
…   hue=“Network”,
… )
>>> plt.xticks(plt.xticks()[0][::10])
>>> plt.show()

**FIGURE 7:**
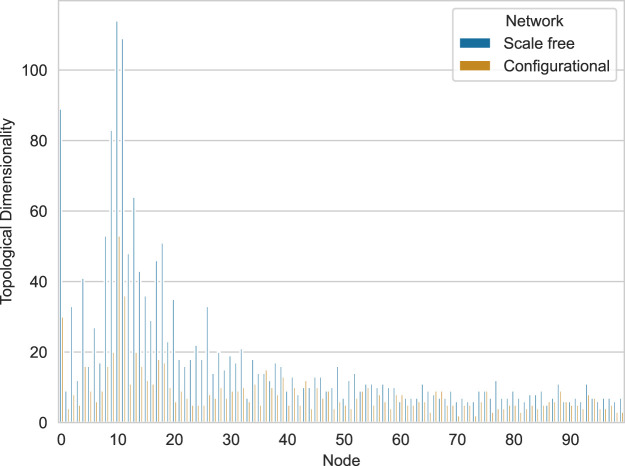
Comparison of topological dimensionality across scale-free and configurational networks with identical degree distributions. Blue bars represent scale-free networks, while orange bars represent configurational networks. x -axis represents nodes, y -axis—topological dimensionality.

The resulting plot is shown in Figure 7. This snippet demonstrates how to compute and visualize topological dimensionality for a pair of networks. Since networks are generated, there is no strict order of nodes and thus it is unreasonable to compute median topological dimensionality for each node. Though such aggregation is possible in case of real networks, where nodes have some sort of ordering (for example, anatomical nodes in the brain), we can compute median topological dimensionality for each network type.

### 3.2 Application of Q-analysis to the DBLP dataset of co-authors

To illustrate how Q-analysis can reveal temporal shifts in collaborative structures, this section examines the Coauthors DBLP (Digital Bibliography and Library Project) co-authorship network across three distinct years: 1987, 2002, and 2017 (Benson et al., 2018). In this dataset, publications and their authors form simplices, with authors as vertices. The analysis uses a reduced version of the dataset, excluding simplices with more than 25 coauthors.

**FIGURE 8:**
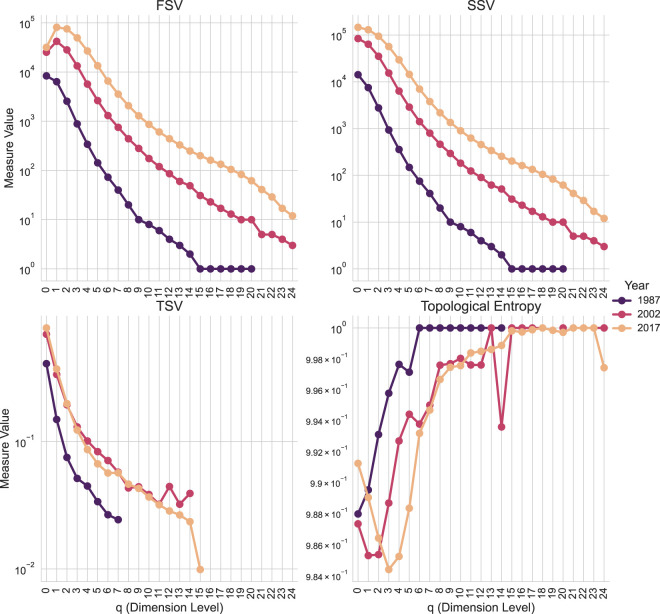
Q-analysis graded parameters of Co-authorship Networks by Year. The plot shows the values of the First Structure Vector (FSV, Q ), Second Structure Vector (SSV, n ), Third Structure Vector (TSV, $\bar{Q}$ ), and Topological Entropy $\left(S_{Q}\right)$ for the years 1987, 2002, and 2017.

We begin by computing the core Q-analysis graded parameters for each year’s co-authorship network. Figure 8 visualizes these sets, offering an initial exploratory view of the evolving topological characteristics of academic collaboration. The following code demonstrates how to generate this data. It involves creating a SimplicialComplex for each year and calling the graded_parameters method. The method returns a GradedParameters object, which can be converted to a pandas DataFrame using its to_dataframe () method. The resulting DataFrame is suitable for direct use with standard Python visualization libraries.

>>> from q_analysis import SimplicialComplex
>>> from q_analysis.datasets import load_dataset
>>> import pandas as pd
>>> simplices_by_year = load_dataset(“coauthors”)
>>> years = [2017, 2002, 1987]
>>> df = pd.concat([
…   SimplicialComplex(simplices_by_year[year])
…     .graded_parameters(
…      desired_vectors=[’fsv’, ’ssv’, ’tsv’, ’entropy’]
…     )
…     .to_dataframe()
…     .assign(Year=year)
…   for year in years
… ], ignore_index=True)

The code computes a reduced set of graded parameters, omitting simplex and shared face counts. These results are visualized in Figure 8 using matplotlib and seaborn.

The analysis shows an increase in both the total number of simplices (papers) and their connected component sizes from 1987 to 2017, reflecting expected growth in research output and collaboration. Analysis of the Third Structure Vector (TSV) reveals distinct temporal dynamics in higher-order connectivity. TSV values for 1987 are generally lower across most q-levels compared to 2002 and 2017, indicating a more fragmented co-authorship landscape in the earlier period. The relative stability and higher values of TSV for 2002 and 2017 suggest a more mature and interconnected network structure. The output highlights features such as minor connectivity peaks in the 2002 TSV profile at q-orders 12 and 14. These patterns suggest specific collaboration structures (chains of 12- or 14-near authors) that were less prevalent in 2017.

The graded_parameters function also computes topological entropy $\left(S_{Q}\right)$ , visualized in Figure 8. The observed shift of the $S_{Q}$ minimum to higher orders from 1987 to 2017 suggests an increased tendency for author groups to collaborate on multiple papers, indicating greater participation diversity. A local minimum in topological entropy for q=14 in 2002 points to the presence of researcher communities of approximately 15 authors (q+1) participating in a few papers simultaneously, a pattern that invites further investigation into the nature of these collaborations.

**FIGURE 9:**
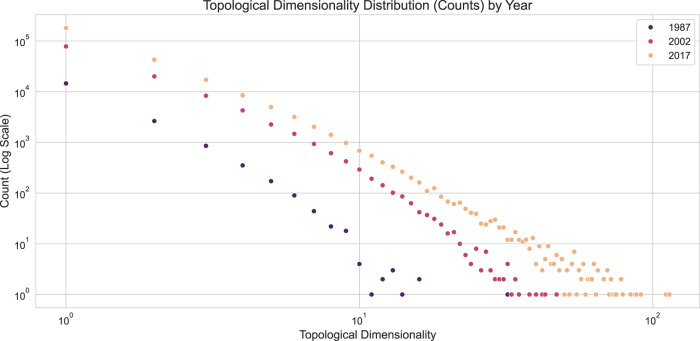
Topological Dimensionality distribution of Co-authorship Networks by Year. The plot shows the count of authors (y-axis, log scale) for each topological dimensionality value (x-axis) for the years 1987, 2002, and 2017.

To explore individual author engagement in the co-authorship structures, topological dimensionality was computed using the topological_dimensionality () method from the SimplicialComplex class. This method returns a NodeParameterSet object containing per-node (author) values, which can be converted to a DataFrame for aggregation and distributional analysis, as shown in Figure 9.

>>> from q_analysis.simplicial_complex import SimplicialComplex
>>> from q_analysis.datasets import load_dataset
>>> import pandas as pd
>>> dataset = load_dataset(“coauthors”)
>>> years = [1987, 2002, 2017]
>>> topological_dimensionality_by_year = pd.concat([
…    SimplicialComplex(dataset[year])
…      .topological_dimensionality()
…      .to_dataframe()
…      .assign(Year=year)
…    for year in years
… ]).groupby([“Topological Dimensionality”, “Year”])\
…    .count()\
…    .reset_index()

The distributions in Figure 9 reveal a power-law trend, a common observation in networks. Since topological dimensionality counts the number of simplices a node participates in, this metric is analogous to the graph-based concept of node degree. However, the two metrics measure different aspects of network structure. To compare them directly, node degrees can be computed from the simplicial complexes. This involves projecting the complex into a graph representation using the SimplexProjection transformer, as shown in the code below, and then calculating the degree for each node in the resulting graph.

>>> simplicial_complexes_by_year = [
…    SimplicialComplex(dataset[year])
…    for year in years
… ]
>>> graph_projector = SimplexProjection(q=0, weighted=True)
>>> projected_complexes = graph_projector.transform(
…   simplicial_complexes_by_year
… )

**FIGURE 10:**
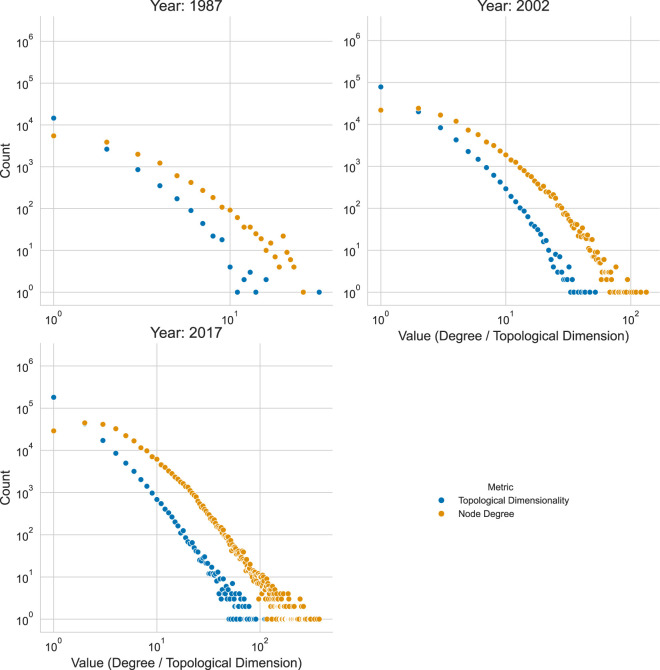
Topological Dimensionality vs. Node Degree Distribution by Year. The plot shows the count of authors (y-axis, log scale) for each topological dimensionality value (x-axis) for the years 1987, 2002, and 2017.

The resulting array holds sparse adjacency matrices. Figure 10 shows the difference between the node degree and topological dimensionality distributions.

### 3.3 Application of Q-analysis to a network physiology: uncovering higher-order brain network disruptions in major depressive disorder

To demonstrate the practical utility of the q-analysis package, we highlight its application in one of our recent studies investigating higher-order brain connectivity in Major Depressive Disorder (MDD) using fMRI data (Kurkin et al., 2024). While traditional analysis focuses on pairwise interactions, this approach often fails to capture the complex, multi-region coordination that underlies brain function. Our package provides the necessary tools to explore these higher-order structures.

In that study, we analyzed fMRI-based ROI-to-ROI functional brain networks from MDD patients (n=70) and healthy controls (HC, n=94 ), reconstructed using Pearson correlation. The ROIs were defined using the AAL3 anatomical brain atlas (Rolls et al., 2020), resulting in 165×165 functional connectivity matrix. For more details, see (Kurkin et al., 2024; Pitsik et al., 2023). Using the q-analysis package, these networks were converted into SimplicialComplex objects by treating maximal cliques as simplices. The graded_parameters method was then employed to compute key Q-analysis metrics, which serve as topological “fingerprints” of the networks.

**FIGURE 11:**
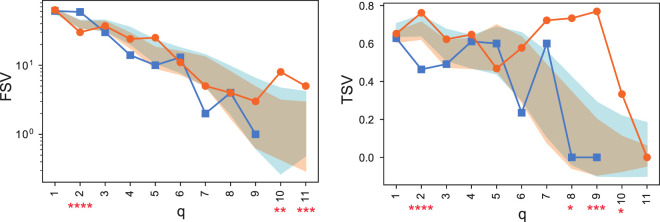
Comparison of the First Structure Vector (FSV, left) and Third Structure Vector (TSV, right) for healthy control (HC, orange) and MDD (blue) consensus networks. The y-axis for FSV is on a logarithmic scale. Shaded areas represent the standard deviation from permutation testing, and asterisks denote statistically significant differences. These vectors were computed using the graded_parameters functionality of the package. Adapted from Kurkin et al. (2024).

The computation of structure vectors, such as the First Structure Vector (FSV) and Third Structure Vector (TSV), revealed significant topological differences (Figure 11).

As seen in Figure 11, the FSV for the MDD group was significantly higher at q=2 , indicating greater fragmentation (more isolated components) at the level of simple pairwise links. Conversely, the HC group’s network retained connectivity at much higher dimensional levels ( q=10 and q=11 ), a feature entirely absent in the MDD networks. The TSV plot reinforces this, showing higher connectivity for the HC group at these advanced q -levels.

**FIGURE 12:**
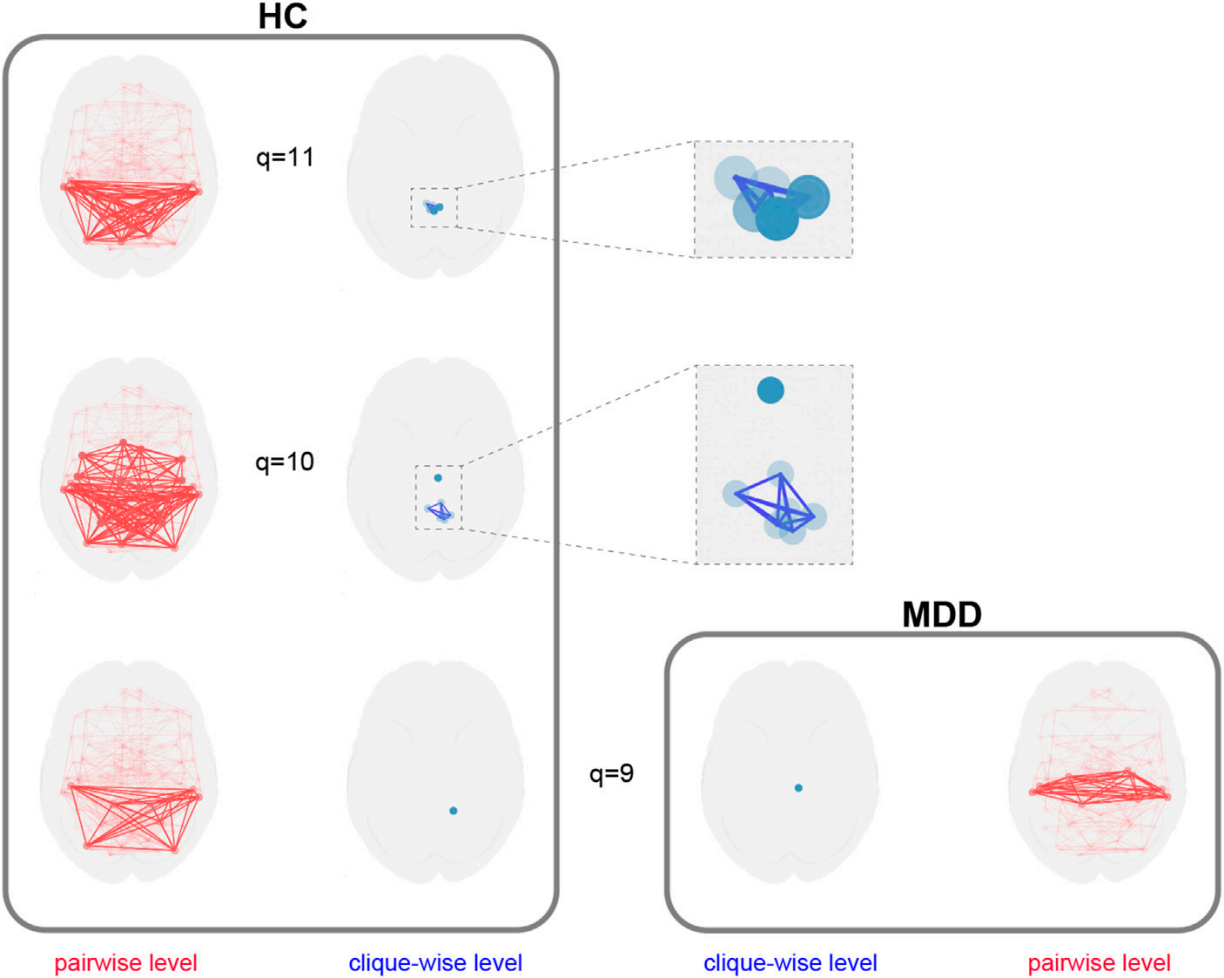
Multilayer decomposition of higher-order network structures. The healthy control (HC) network contains distinct q-connected components at q=10 and q=11 , representing complex, multi-region functional hubs. In contrast, the MDD network has no components beyond q=9 , indicating a loss of these higher-order integrative structures. Adapted from Kurkin et al. (2024).

These quantitative differences motivated a deeper, qualitative exploration of the network structure at high q -levels. To visualize the underlying structures driving these differences, we used the package to identify and analyze the *q-connected components* for q≥9 . The results are depicted in Figure 12.

The HC network decomposes into multiple, distinct higher-order components at q=10 and q=11 , reflecting a rich and diverse topological organization. The MDD network, however, contains no structures beyond q=9 , where it consists of only a single, isolated component. This illustrates a profound disruption in the brain’s ability to form highly integrated higher-order functional assemblies in MDD.

This application demonstrates a workflow for analyzing higher-order network structures in the brain. First, the package is used to compute quantitative metrics like structure vectors, which identify topological differences between groups (Figure 11). Subsequently, the specific q-connected components underlying these differences are extracted for qualitative analysis and visualization (Figure 12).

The findings suggest the potential of Q-analysis metrics as candidate biomarkers for MDD. Traditional graph theory analyzes pairwise connections, whereas Q-analysis quantifies the integrity of multi-region functional assemblies. This approach complements standard methods by linking local connectivity deficits to changes in large-scale network organization, providing a different perspective on brain topology in disease.

## 4 Summary and discussion

This paper introduces q-analysis, a Python package for performing Q-analysis on complex networks. We present the mathematical background of Q-analysis and detail the package’s implementation of its core metrics, such as structure vectors and topological entropy. The package provides tools for constructing simplicial complexes and computing a suite of measures that serve as quantitative summaries of higher-order network structure. By making these metrics accessible and formatting them in standard data structures, the package facilitates the characterization and comparison of network topologies, bridging the gap between the theoretical framework of Q-analysis and practical data analysis workflows. Core computational routines are implemented in Rust for high performance.

The package’s utility is demonstrated through a simulation study comparing scale-free networks to configurational networks with identical degree distributions. The results show how Q-analysis metrics reveal significant differences in higher-order topology that are not apparent from degree distributions alone. Scale-free networks exhibit connectivity concentrated in lower-dimensional structures, whereas configurational networks show more evenly distributed connectivity across dimensions.

Furthermore, we applied the package to a real-world DBLP co-authorship dataset, analyzing the evolution of its collaborative structures over 3 decades (1987, 2002, and 2017). This analysis uncovered temporal shifts in research collaboration, including an increase in the number and size of connected components, changes in higher-order connectivity patterns revealed by the Third Structure Vector, and evolving author participation diversity measured by topological entropy. We also found that topological dimensionality, a higher-order analogue to node degree, follows a power-law distribution, and we demonstrated how it provides a different perspective on node importance compared to traditional degree centrality.

Finally, we demonstrated how to use the package to study a physiological problem by identifying disruptions in the fMRI-derived brain network caused by major depressive disorder. Our analysis revealed significant alterations in the topology of brain networks in MDD patients, characterized by a lower maximum topology level and an increased prevalence of isolated edges and chains at the pairwise interaction level. Additionally, we identified significant disruptions in the higher-order organizational structures of the brain, characterized by reduced topological diversity and complexity, fewer and less connected cliques, and altered involvement of key brain regions in MDD.

While Q-analysis was originally designed for naturally occurring simplicial complexes, it can also be applied to analyze higher-order structures (cliques) within traditional graphs. Interpreting cliques as simplices offers a different perspective on network topology. The package handles both naturally occurring simplicial complexes and those derived from graph clique decompositions. The Q-analysis metrics, such as structure vectors and topological entropy, provide a concise representation of network structure. They capture topological features that allow for analysis and comparison across different networks.

In neuroscience, for instance, Q-analysis could be applied to several open problems. It could be used to track the dynamic reconfiguration of multi-region functional assemblies during cognitive tasks, or to characterize topological changes in brain networks across the lifespan. By quantifying the structure of these complex interactions, the framework offers a path toward developing more sensitive biomarkers for neurological and psychiatric disorders, moving beyond pairwise connectivity to capture the collective behavior of brain circuits.

The Q-analysis framework, as implemented in this Python package, has potential for future extensions. Further development could explore the new metrics, algorithms, and visualization techniques, potentially leading to additional insights into complex systems. The open-source nature of the package is intended to encourage contributions and further development.

## Data Availability

Publicly available datasets were analyzed in this study. This data can be found here: https://github.com/pakrentos/q-analysis.
